# Magnesium in the treatment of alcohol withdrawal syndrome: a multicenter randomized controlled trial

**DOI:** 10.1093/alcalc/agad040

**Published:** 2023-06-13

**Authors:** Donogh Maguire, Donald McMillan

**Affiliations:** Emergency Medicine Department, Glasgow Royal Infirmary, 84 Castle Street, Glasgow G4 0SF, United Kingdom; Academic Unit of Surgery, School of Medicine, University of Glasgow, New Lister Building, Royal Infirmary, Glasgow G31 2ER, United Kingdom; Academic Unit of Surgery, School of Medicine, University of Glasgow, New Lister Building, Royal Infirmary, Glasgow G31 2ER, United Kingdom

## Dear Editor

We read ‘Magnesium in treatment of alcohol withdrawal syndrome: a multicenter randomised controlled trial’ by Airagnes and co-workers (Airagnes *et al*. 2023) with some interest. In particular, from this trial, it would appear that magnesium supplementation is of little clinical benefit in the treatment of patients with Alcohol Withdrawal Syndrome (AWS). These results are in contrast to our recent single blind randomised controlled trial of intravenous magnesium +/− intravenous thiamine in a cohort of patients presenting to an inner-city hospital Emergency Department with AWS (Maguire *et al*. 2022). In the Maguire trial, there was earlier resolution of the AWS score among patients who received magnesium sulfate. The basis of the difference in clinical outcomes between the trials is not clear. However, the dose of magnesium administered to patients and route of administration were different between the Airagnes and Maguire trials (426 mg orally per day for 3 days vs 2.0 g intravenously over 30 minutes, respectively). Also, the proportion of patients who could be considered magnesium deficient (serum magnesium <0.75 mmol/L) was different between the Airagnes and Maguire trials (23 vs 59%, respectively). Furthermore, no significant change in serum magnesium concentrations were achieved between placebo and intervention arms in the Airagnes trial, whereas a significant increase in serum magnesium concentrations was achieved in the Maguire trial. Finally, baseline data indicate that the majority of patients in the Airagnes trial were experiencing mild–moderate AWS (mean CIWA-Ar = 12) at the time of recruitment whereas the majority of patients in the Maguire trial (55%) were experiencing severe AWS at time of recruitment to the trial (Modified Glasgow Alcohol Withdrawal Score ≥ 4). Therefore, further research on the effect of magnesium supplementation on clinical outcomes in patients with AWS is warranted.
